# Severe Altered Immune Status After Burn Injury Is Associated With Bacterial Infection and Septic Shock

**DOI:** 10.3389/fimmu.2021.586195

**Published:** 2021-03-02

**Authors:** Hélène Moins-Teisserenc, Debora Jorge Cordeiro, Vincent Audigier, Quentin Ressaire, Mourad Benyamina, Jérome Lambert, Guitta Maki, Laurence Homyrda, Antoine Toubert, Matthieu Legrand

**Affiliations:** ^1^Université de Paris, Paris, France; ^2^Assistance Publique – Hôpitaux de Paris (AP-HP), Paris, France; ^3^INSERM UMR-1160, Institut de Recherche Saint-Louis, Paris, France; ^4^Biological Haematology Laboratory, Saint-Louis Hospital, Paris, France; ^5^Immunology-Histocompatibility Laboratory, Saint-Louis Hospital, Paris, France; ^6^CEDRIC EA 4629, MSDMA, CNAM, Paris, France; ^7^Department of Anesthesiology and Critical Care and Burn Unit, Saint-Louis Hospital, Paris, France; ^8^Département of Biostatistics, Saint-Louis Hospital, Paris, France; ^9^Department of Anesthesiology and Perioperative Care, University of California, San Francisco, San Francisco, CA, United States

**Keywords:** burns, immunosuppression, inflammation, outcome, prognostic, intensive care

## Abstract

**Introduction:** Burn injury is associated with a high risk of death. Whether a pattern of immune and inflammatory responses after burn is associated with outcome is unknown. The aim of this study was to explore the association between systemic immune and inflammatory responses and outcome in severely-ill burn patients.

**Materials and Methods:** Innate immunity, adaptive immunity, activation and stress and inflammation biomarkers were collected at admission and days 2, 7, 14, and 28 in severely-ill adult burn patients. Primary endpoint was mortality at day 90, secondary endpoint was secondary infections. Healthy donors (HD) served as controls. Multiple Factorial Analysis (MFA) was used to identify patterns of immune response.

**Results:** 50 patients were included. Age was 49.2 (44.2–54.2) years, total burn body surface area was 38.0% (32.7–43.3). Burn injury showed an upregulation of adaptive immunity and activation biomarkers and a down regulation of innate immunity and stress/inflammation biomarkers. High interleukin-10 (IL-10) at admission was associated with risk of death. However, no cluster of immune/inflammatory biomarkers at early timepoints was associated with mortality. HLA-DR molecules on monocytes at admission were associated with bacterial infections and septic shock. Later altered immune/inflammatory responses in patients who died may had been driven by the development of septic shock.

**Conclusion:** Burn injury induced an early and profound upregulation of adaptive immunity and activation biomarkers and a down regulation of innate immunity and stress/inflammation biomarkers. Immune and inflammatory responses were associated with bacterial infection and septic shock. Absence of immune recovery patterns was associated with poor prognosis.

## Introduction

Severe burn injury is one of the most life-threatening traumas. After burn injury, peripheral tissues release multiple proinflammatory mediators, reactive nitrogen and oxygen species, causing a post-burn systemic inflammatory response syndrome (1, 2). Under physiological conditions, three major defense mechanisms protect individuals from infections: skin and mucosal barriers and mediators of innate and adaptive immune responses (3). The dysfunction of the immune system is a hallmark of critically-ill patients. It is now well-established that both pro-inflammatory and anti-inflammatory responses may occur at early time points of sepsis, trauma or burn injury (4, 5). An elevation of serum cytokine level has been repeatedly reported after burn injury. Gauglitz et al. reported that pediatric burn patients with increased IL-6 and IL-10 as well as decreased IL-7 serum levels after inhalation injury had significantly greater mortality risk (6). The intensity of the inflammatory response appears associated with the total burn surface area. Finnerty et al. reported higher IFN-γ, IL-10, IL-17, IL-4, IL-6, and IL-8 levels in adults compared with children during the first week following burn injury (7). Furthermore, differences between the immune response to sepsis and burn injury patients have been highlighted. Burn injury pediatric patients dying of sepsis developed inflammatory profiles significantly different from those of non-septic counterparts (7). Burn injury has further been associated with immunosuppression, including apoptosis-induced lymphopenia and decreased monocyte human leukocyte antigen-DR (mHLA-DR) (8). In a single center study, persistent decrease of mHLA-DR expression was associated with mortality and the development of infectious complications (9). Altogether burn injury has been shown to be associated with systemic immune response, elevated serum cytokine levels and immunosuppression. How the different biomarkers interact with each other and associate with outcomes is however still poorly understood. During the last decade, progress in critical care and burn care has led to major improvement in burn injury patient outcomes. Nonetheless, these patients are at high risk of developing infections and sepsis, accounting for the majority of deaths. The objective of the current study was to profile the host immune response to burn injury in critically-ill patients and explore potential clusters associated with outcomes.

## Materials and Methods

### Patients

Patients older than 18 years with deep burn total body surface area (TBSA) >20% or TBSA >15% together with organ failure (i.e., requiring invasive mechanical ventilation and/or vasopressors) admitted to a referral burn center in France were included between October 2013 and February 2016. Blood samples were collected on the day of admission (D0), Day 2 (D2), Day 7 (D7), Day 14 (D14), and Day 28 (D28) after admission for analysis of immune and inflammatory response biomarkers (Supplementary Table 1). Samples from HD collected from the blood donor center (Etablissement Français du Sang, Saint-Louis hospital) served as controls. This study was approved by our local ethical committee (IRB 00003835, protocol 2013/17NICB) and was in accordance to the Declaration of Helsinki.

The following information was collected: demographic characteristics: age, sex, height, weight, comorbidities and previous treatments, admission characteristics TBSA, smoke inhalation injury, Simplified Acute Physiology Score II - SAPS II, Abbreviated Burn Severity Index (ABSI), treatments, causative pathogens, renal outcome, organ supports and 90-day mortality (Table 1).

**Table 1:** Clinical characteristics of patients.

	**All patients (*n* = 50)**	**Alive at D90 (*n* = 35)**	**Dead at D90 (*n* = 15)**
Male (*n*/%)	30 (60.0%)	24 (68.6%)	6 (40.0%)
Age (years)	49.2 (44.2–54.2)	43.3 (37.1–49.5)^*^	53.8 (47.0–60.6)^*^
**Comorbidities (*****n*****/%)**			
Auto-immunity	3 (6.0%)	3 (8.6%)	0 (0.0%)
Tobacco use	17 (34.0%)	12 (34.3%)	5 (33.3%)
Alcoholism	13 (26.0%)	9 (25.7%)	4 (26.7%)
COPD	3 (6.0%)	2 (5.7%)	1 (6.7%)
High Blood Pressure	13 (26.0%)	6 (17.1%)^*^	7 (46.7%)^*^
Diabetes	6 (12.0%)	4 (11.4%)	2 (13.3%)
Cirrhosis	1 (2.0%)	0 (0.0%)	1 (6.7%)
Stroke	5 (10.0%)	2 (5.7%)	3 (20.0%)
BMI (kg/m^2^)	26.5 (24.4–28.6)	26.0 (23.6–28.3)	29.2 (24.6–33.8)
**Burn Injury**			
TBSA (% of TSA)	38.0 (32.7–43.3)	33.0 (29.1–36.9)^*^	51.5 (37.7–65.3)^*^
Depth (% of TBSA)	52.4 (42.1–62.6)	46.2 (33.7–58.6)	67.1 (49.1–85.2)
Inhalation (*n*/%)	17 (34.0%)	9 (25.7%)	8 (53.3%)
Closed room (*n*/%)	28 (56.0%)	19 (54.3%)	9 (60.0%)
Thermal (*n*/%)	47 (94.0%)	32 (91.4%)	15 (100.0%)
Electric (*n*/%)	3 (6.0%)	3 (8.6%)	0 (0.0%)
**Initial care**
Hydroxobalamin (*n*/%)	5 (10.0%)	2 (5.7%)	3 (20.0%)
Intubation (*n*/%)	28 (56.0%)	17 (48.6%)	11 (73.3%)
Amines (*n*/%)	4 (8.0%)	2 (5.7%)	2 (13.3%)
Sedations (*n*/%)	31 (62.0%)	19 (54.3%)	12 (80.0%)
Incisions (*n*/%)	18 (36.0%)	10 (28.6%)	8 (53.3%)
**Severity scores**			
UBS	90.5 (66.6–114.4)	90.0 (72.7–107.3)^*^	148.5 (85.8–211.2)^*^
ABSI	8.0 (7.2–8.8)	8.0 (7.3–8.7)^*^	10.0 (8.5–11.5)^*^
SAPS2	35.0 (30.4–39.6)	27.5 (22.8–32.2)^*^	40.5 (31.9–49.1)^*^
Length of stay (days)	38.5 (30.6–46.4)	44.0 (34.7–53.3)^*^	23.0 (12.0–34.0)^*^
RRT (*n*/%)	13 (26.0%)	3 (8.6%)^*^	10 (66.7%)^*^
**Infection (*****n*****/%)**			
Bacterial	39 (78.0%)	26 (74.3%)	12 (80.0%)
Viral	19 (38.0%)	13 (37.1%)	5 (33.3%)
Fungal	12 (24.0%)	7 (20.0%)	5 (33.3%)
Septic shock	19 (38.0%)	7 (20.0%)^*^	12 (80.0%)^*^
**Surgery (*****n*****/%)**			
Autograft	35 (70.0%)	28 (80.0%)^*^	7 (46.7%)^*^
Allograft	8 (16.0%)	4 (11.4%)	4 (26.7%)
Xenograft	10 (20.0%)	6 (17.1%)	4 (26.7%)

* *p < 0.05*.

*COPD, Chronic Obstructive Pulmonary Disease; BMI, Body Mass Index; TBSA, Total Burn Skin Area; TSA, Total Skin Area; UBS, Skin Burn Unit; ABSI, Abbreviated Burn Severity Index; SAPS2, Simplified Acute Physiology Score; RRT, Renal Replacement Therapy*.

### Study Endpoints

The primary endpoint measure was death 90 days after admission. Secondary endpoints were occurrence of secondary infections (bacterial, fungal and viral infections) and septic shock. Septic shock was defined as an infection with persistent hypotension despite adequate fluid resuscitation and/or Lactate >4 mmol (10). All episodes of infection (including source and causative pathogen-s) were prospectively validated in weekly multidisciplinary staff meetings including intensivists, infectious disease specialists, surgeons and microbiologists. Invasive fungal infection (IFI) was defined following adaptation of the European Organization for Research and Treatment of Cancer (EORTC) and the Mycoses Study Group EORTC/MSG criteria (11). Proven or probable IFI was defined if evidences of any of the following were observed: (i) vascular invasiveness and tissue invasion upon histological examination; (ii) clinical necrosis and repeated positive mycology; and/or (iii) positive blood culture to fungi. Viral infections were defined as either positive lung and/or plasma sampling with >4.5 log of viral load with clinical symptoms or organ failure. Patients were treated with intravenous acyclovir for herpes-related infections or ganciclovir for cytomegalovirus-related infections.

### Flow Cytometry and Cytokine Assay

Immunostainings were performed on freshly collected (Na_2_EDTA tubes) whole blood samples, using a FACS Canto II flow cytometer and FACS DIVA software (BD Biosciences), in a laboratory that operates under principles of Good Laboratory Practice (GLP). Absolute counts were determined using the TruCount system (BD Biosciences) with anti-CD3, -CD8, -CD45, and -CD4 mAbs (BD Multitest, BD Biosciences). Biomarkers were clustered into four predefined subsets (i.e., innate and adaptive immunity activation, stress/inflammation) (Supplementary Table 1). Eight color staining was performed with the following mAbs (all from BD unless specified) to assess (i) innate immunity [Polynuclear cells (PN), NK, iNKT, monocytes, MAIT, γδ T-cells]: anti-CD45 (FITC, PerCP, v500), -CD14 (PerCP), -CD56 (PE-Cy7), -CD16 (APC-H7), -CD57 (FITC), -CD64 (PE), -NKG2D (PE), -NKp30 (APC), -TCR γδ (FITC), -Vα7.2 (APC), -CD161 (BV421), -iNKT [TCRVα24-Jα18, (PE)], and -CD274/PDL1 (Pe-Cy7); (ii) “stress” responses: anti-MICA (FITC), -B7H6 (APC) (kindly provided by Eric Vivier, Marseille); (iii) adaptive immunity (T and B-cells): anti-CD45RA (APC), -CD3 (PE-Cy7, APC-H7, v450), -CD4 (v500), -CD8 (PerCP), -CCR7 (BV421), -CD27 (APC-H7), -CD28 (PE), -CD25 (PE-Cy7), -HLA-DR (APC-H7), -CD279/PD1 (PE), -CD19 (PE-Cy7), -IgD (FITC), -CD10 (APC), and -CD38 (v450); and (iv) the number of HLA-DR molecules per monocyte (HLA-DR/monocyte) after calibration with Quantibrite™ (BD Biosciences), as previously described (12). Of note, Mucosal Associated Invariant T (MAIT) cells were defined as CD3+CD4-γδ-CD161^hi^Vα7.2+ (13). The level of non-specific background signals on monocytes was appreciated with isotype controls and results were expressed according to the ≪ fluorescence minus one ≫ (FMO) control. FACS data were analyzed as percentage of parental subsets. The level of cell-surface expression was assessed by the Mean Fluorescence Intensity (MFI) ratio between positive and negative subset as well as the stain index (SI) that normalizes the positive signal to the unstained background (Supplementary Table 1). Standardization was performed using 8-peak Rainbow beads (BD Biosciences). The Cytometric Bead Array (CBA) assay (BD Biosciences) enabled multiplex analysis of the plasma cytokines: IL-2, IFN-γ, IL-4, IL-6, IL-10, IL-17, and TNF-α. Data were analyzed using FlowJo® software. Flow Cytometry hierarchical gating strategy is described in Supplementary Figure 1, PN, monocytes and lymphocytes were defined using CD45 and morphological criteria. Representative examples are shown in Supplementary Figure 2 (HD) and Supplementary Figure 3 (patients and HD).

### Statistical Analysis

Biomarker data were compared between survivors and non-survivors at day 90 and between burn injury patients with or without infections using a Kruskall Wallis test with multiple comparisons. Multiple Factorial Analysis (MFA) was used to analyze the variability between immune profiles with the FactoMineR package from the R software (14–16).

MFA is an extension of principal component analysis (PCA) achieving the same goals but allows the balance of each group of biomarkers in the calculation of dissimilarities between immunologic profiles, as a standardization in PCA allowing balance between continuous variables. First, MFA was used to explore immune profiles differences between burn injury patients and healthy controls. Analysis was based on 69 descriptors (i.e., biomarkers) common to both groups. Variables with missing values over than 60% were omitted. Missing values were handled by iterative MFA (17). Wilcoxon tests were applied to exhibit the statistical significance of the mean coordinates of burns and controls on the first dimensions. Then, MFA was used to explore immune profiles of burn injury patients at admission and at D7. The analysis was based on 84 descriptors and a dataset of 43 individuals. The analysis of the relationships between all variables and factors was based on correlation circles. Wilcoxon tests were applied to compare the mean position of individuals on the first dimension according to the outcomes of interest. We excluded patients who died within the first 7 days from admission when investigating the association between immune profile and mortality, as deaths before D7 may be related to uncontrolled multiple organ failure or a decision to withdraw life support. Finally, MFA was used to explore differences between immune profile trajectories of burn injury patients between admission and D7 for immune recovery. MFA was performed at admission and values at the seventh day were added. For each patient, coordinates at baseline and D7 defined trajectories clustered in two groups by k-means clustering which is a distance-based method, assessing proximities and differences between objects (variables or individuals), according to Euclidean distance.

Fisher exact tests were performed to compare the association between outcomes and groups of immune profile trajectories between admission and D7. Based on the above-mentioned rules, the analysis focused on 39 individuals and 84 descriptors. As a sensitivity analysis, biomarkers were separated in activation, adaptive, innate and stress/inflammation categories.

## Results

### Patient Clinical Characteristics

Fifty severely-ill burn patients were included. Median age was 49.2 (44.2–54.2) years, 60% were male, Abbreviated Burn Severity Index (ABSI) was 8.0 (7.2–8.8) and simplified acute physiology score (SAPS2) was 35.0 (30.4–39.6). Median total burn body surface area was 38.0% (32.7–43.3) (Table 1). Thirty-eight patients (76%) received invasive mechanical ventilation. Thirteen patients (26%) required a renal replacement therapy (RRT) during their ICU stay.

### Mortality

Eight patients (16%) died before D28 and 15 between D28 and D90 (30%). Patients who died before D90 had a significantly shorter stay in the intensive care unit (23.0 vs. 44.0 days, *p* < 0.005), were more likely to develop septic shock (80.0 vs. 20.0%, *p* < 0.001) and acute kidney injury, and required more frequently renal replacement therapy (66.7 vs. 8.6%, *p* < 0.0001).

### Acquired infections

During hospitalization, 40 (80%) patients had at least one documented infection. Thirty-nine patients developed a bacterial infection (78%), 12 patients (24%) developed IFI, including eight with a filamentous pathogen and four patients (44%) with *Candida* sp. Nineteen patients (38%) developed a septic shock during ICU stay, of which all had a bacterial infection, 67% had an IFI and 58% had a viral infection (Table 1).

Patients who developed an infection had a longer length of stay (44.0 vs. 23.0, *p* < 0.005). Incidence of bacterial infections, IFI or viral infections was not statically different between survivors (S) and non-survivors (NS) at D90 (S:74.3 vs. NS:80 %; S:20 vs. NS:33.3%; S:37.1 vs. NS:33.3%, respectively, in cases of bacterial, fungal or viral infections).

### Burn Injury Induced Early and Profound Immunological and Inflammatory Response

**Figure 1:**
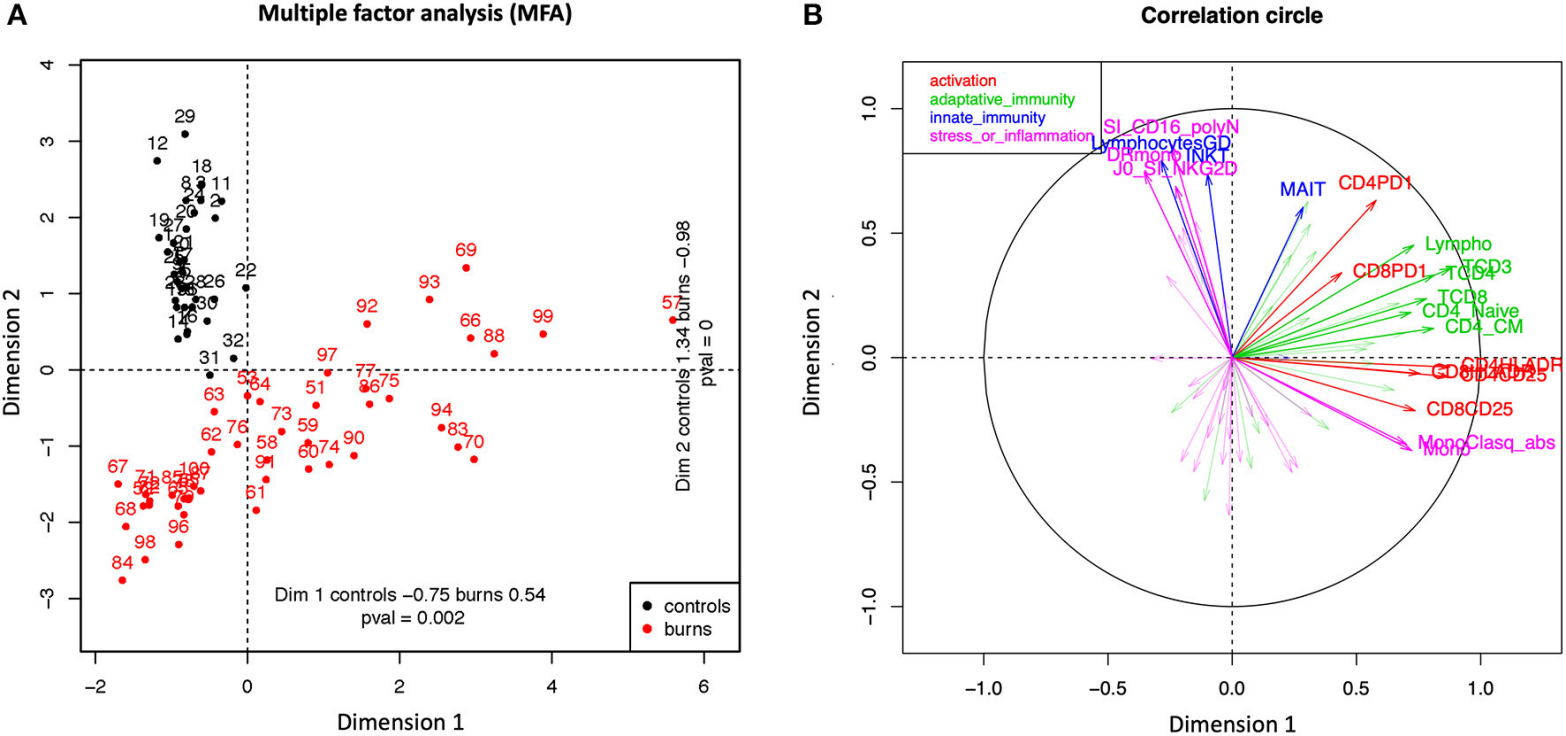
Multiple Factor Analysis (MFA). The analysis was based on 69 biomarkers and 76 individuals (healthy volunteers and burn patients at D0). **(A)** Healthy Donors (black dots) and burn patients (red dots) are presented as points on the scatter plot created with the first two main dimensions of Multiple Factorial Analysis (MFA). This analysis displayed the variability within the group of burn patients through dimension 1 (index of repartition: 0.54) whereas all controls are less spread in dimension 1 (controls: 0.75). **(B)** The correlation circle was generated using the most discriminating biomarkers between the two groups of individuals. The biomarkers were classified in four groups: activation markers (red), adaptive immunity markers (green), innate immunity markers (blue) and stress or inflammation markers (pink).

MFA was performed on 69 descriptors common to both HD and patient groups at D0. Only individuals with more than 30% of observed values were kept in the MFA, leading to a dataset of 76 observations. Figure 1A represents the first individual factor map with HD in black and burn patients in red. Differences between coordinates on the first and second axes (horizontal and vertical, respectively) are explained by the most correlated variables with the principal component (see correlation circle in Figure 1B or Table 2 and Supplementary Table 2 for listing). Two sets of correlated variables can be distinguished. The first set gathers variables correlated with the first principal component. It consists mostly of biomarkers of adaptive immunity and activation. Biomarkers with a correlation ≥0.6 are listed in Table 2 (see Supplementary Table 2 for more biomarkers ordered by increasing values). All correlations are positive meaning that the largest these variables are, the largest coordinates of individuals on the first axis are. Some burn injury patients aligned with healthy volunteers on component 1, illustrating overlapping adaptive immunity response between some patients and controls. However, they differed on component 2 illustrating that patients and controls did not co-clustered regarding their global immune response. Therefore, immediately after burn injury, the patient status illustrates an enhanced adaptive immune profile with elevated activated markers. A second set of variables is comprised in the second component, including mostly biomarkers of innate immunity, stress and inflammation, all with positive correlation. Burn injury patients are characterized by lower values of circulating innate cells with low expression of inflammatory and stress molecules. Both groups of biomarkers are orthogonal meaning that both tend to be globally unrelated.

**Table 2:** Top biomarkers with a correlation >0.6 in dimension 1 and 2 as analyzed/cited in Figure 1B between burn patients and healthy donors.

**Dimension 1**		**Dimension 2**	
**Biomarker**	**Correlation**	**Biomarker**	**Correlation**
CD3+ T-cells/μL	0.92	SI CD16 Neutrophiles	0.76
HLA-DR+CD4+Tcells/μL	0.82	γδ T-cells/μL	0.76
CD25+CD4+T-cells/μL	0.82	Number of HLA-DR molecules/monocytes	0.72
CD4+T-cells/μL	0.81	iNKT-cells/μL	0.62
CD8+T-cells/μL	0.78	SI NKG2D NK-cells	0.6
CD4+CM T-cells/μL	0.76	CD4+ EM T-cells/μL	0.6
Total Lymphocytes/μL	0.73		
HLA-DR+CD8+Tcells/μL	0.70		
CD25+CD8+T-cells/μL	0.69		
CD4+Naive T-cells /μL	0.67		
RTE/μL	0.66		
B-cells/μL	0.65		
Naive CD8+ T-cells/μL	0.65		
Classical monocytes %/ monocytes	0.60		

Coordinates of HD and burn patients are statistically significant on both axes (*p* < 0.01), meaning that both groups are globally different based on the two sets of variables: burn injury patients have a positive coordinate on the first component and negative for the second one, while the contrary is observed for HD patients (Figures 1A,B and Table 2). In other words, burn patients are mostly identified with biomarkers of adaptive immunity and activation while HD with biomarkers of innate immunity and stress or inflammation biomarkers. Furthermore, little variability is observed between coordinates of HD according to the first axis, while coordinates considerably vary between burn injury patients, highlighting heterogeneity in the immune and inflammatory response in this group. In respect to the second set of biomarkers, large (rather than small) values are specific to HD compared to burn patients (Figure 1B and Table 2).

**Figure 2:**
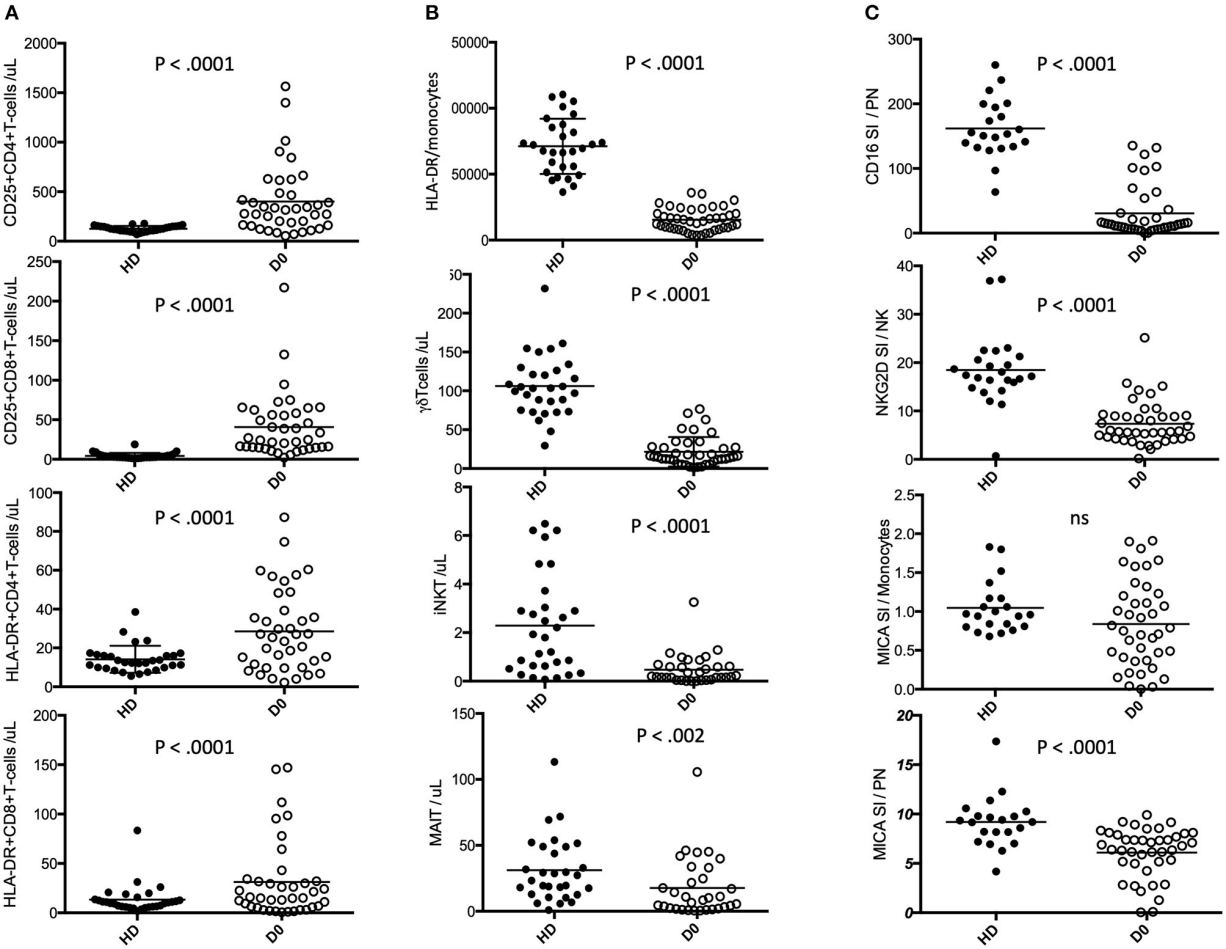
Comparative plots of the selected most discriminative biomarkers. Healthy donors (HD, filled circles) and burn patients at day 0 (D0, open circles) within the group of discriminative activation biomarkers **(A)**. the group of discriminative innate biomarkers **(B)** and the group of discriminative stress and inflammation biomarkers **(C)**.

The MFA results are confirmed in single variable analyses (Figure 2), with burn injury patients and HD showing statistically significant differences in biomarkers of adaptive and innate immunity. Burn patients displayed increased absolute counts of activated CD25+CD4+, HLA-DR+CD4+, CD25+CD8+ and HLA-DR+CD8+ T-cells (Figure 2A) and decreased absolute counts of unconventional lymphocytes such as γδ-T-cells, iNKT, and at a lesser extend Mucosal Associated Invariant T-cells (MAIT) (Figure 2B and Table 2 and Supplementary Table 2). A drastic decrease in the number of HLA-DR molecules per monocyte (HLA-DR/monocytes) was observed during the first hours after burn injury (Figure 2B), with increased monocyte counts (Table 2). Neutrophils expressed lower amounts of the CD16 (FCgRIII receptor), linked to antibody associated cytotoxic cell death (ACCD) (Figure 2C). Similarly, NKG2D expression was dramatically reduced on NK cells. This receptor promotes NK cell functions by triggering stress inducible ligands such as MICA. As opposed to HDs, MICA ligand displayed very heterogeneous levels of expression on patient monocytes. In addition, we observed an unexpected low expression of MICA on patient-derived neutrophils (Figure 2C). Finally, serum levels of the pro-inflammatory cytokines IL-6 and IL-17 as well as the anti-inflammatory cytokine IL-10 were dramatically increased during the first hours after burn injury (Table 3).

**Table 3:** Main serum cytokine level at D0.

**Cytokines**	**Severe burn patients (*n* = 41)**	**Controls (*n* = 10)**	***p***
IL-6 (pg/mL)	1,400 (204.1–2,596)	0.28 (0.02–0.53)	<0.0001
IL-17 (pg/mL)	6.49 (3.58–9.39)	0.93 (0–2.80)	0.02
IL-10 (pg/mL)	56.21 (0–116.6)	0.02 (0–0.06)	<0.0001
IL-4 (pg/mL)	0.3 (0.14–0.47)	0.02 (0–0.05)	<0.01
IL-2 (pg/mL)	0.24 (0.01–0.47)	0.55 (0.02–1.07)	0.02
IFN-γ (pg/mL)	0.24 (0.12–0.36)	0 (0–0)	–
TNF-α (pg/mL)	0.57 (0.11–1.02)	0 (0–0)	–

### Association Between Immune Profile and Mortality

**Figure 3:**
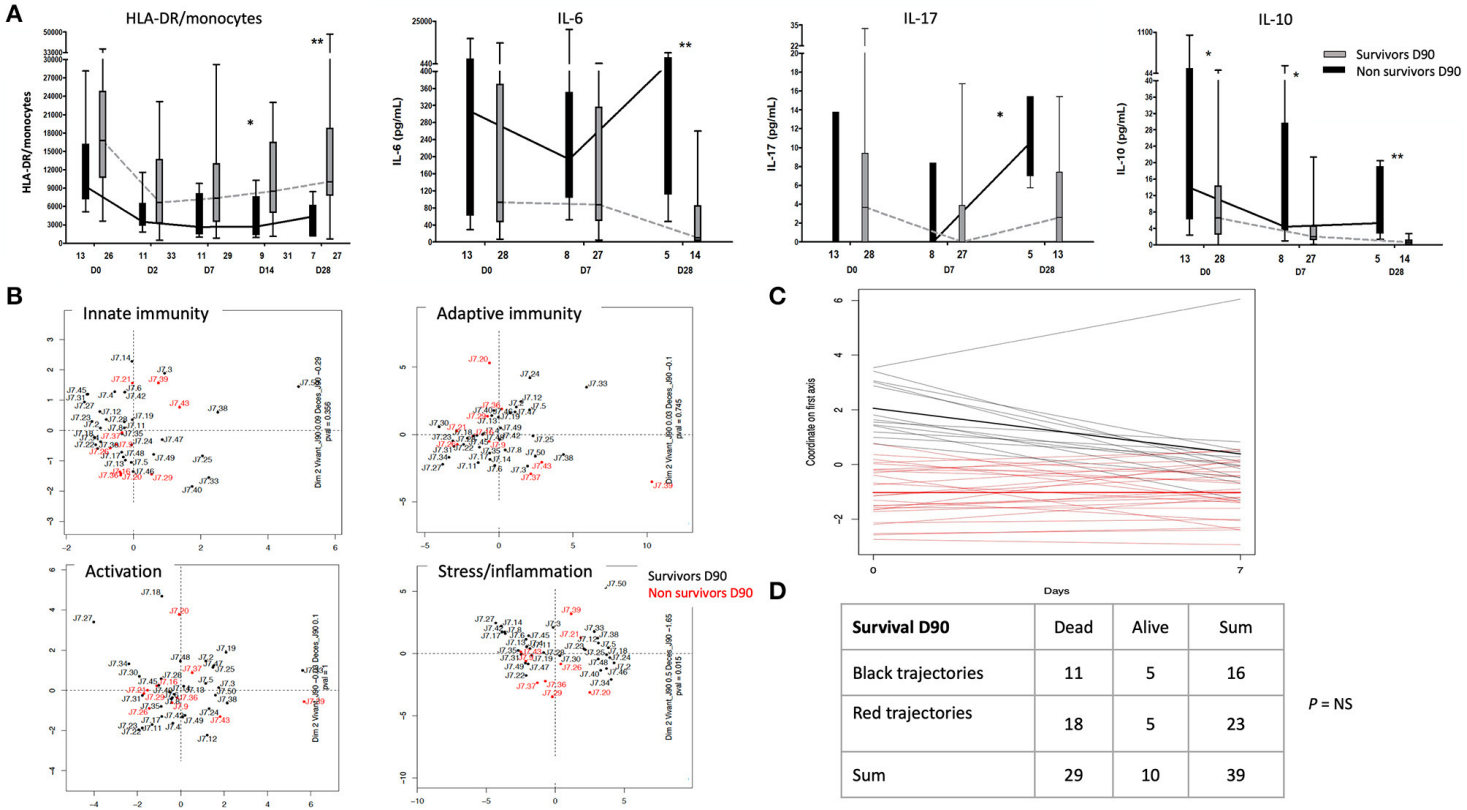
Association between immune profiles and mortality. **(A)** Patterns of monocytes HLA-DR and serum cytokine levels between survivors (in gray) and non-survivors (in black) at day 90. Comparison of the number of HLA-DR molecules per monocyte (HLA-DR/monocytes), IL-10, IL-6, and IL-17 from day (D) 0 to D28 between burn patients who survived at day 90 or not. The number of patients is indicated below each plot. **(B)** Survivors (black dots) and non-survivors (red dots) at day 90 are presented in the first two dimensions of MFA of each four predefined subsets of immune markers. **(C)** Comparative trajectories from D0 to D7. Two classes of trajectories, red and black, were defined by k-means clustering. **(D)** Contingency table displaying patients with red or black trajectory depending on whether they survive at D90. *p < 0.05; ** *p* < 0.001.

The number of HLA-DR/monocytes was not significantly lower during the first week in non-survivors, compared to survivors during the first week (Figure 3A), yet this decreased at D14 and D28, significantly. Serum cytokine levels showed significantly higher IL-10 at admission in non-survivors, which were not correlated with HLA-DR/monocytes (data not shown). Other serum cytokine levels showed no significant difference between survivors and non-survivors at admission, yet non-survivors had higher IL-6, IL-10 and IL-17 at later timepoints during their ICU stay (Figure 3A).

MFA was used to explore differences between immune profiles and identify potential clusters of patients at admission and D7. This analysis was based on 84 descriptors to include 43 individuals. Our data revealed that no cluster of immune biomarkers evaluated at admission or D7 was predictive for survival at D90 (Supplementary Figure 4A, data at D7). When biomarkers were clustered into 4 predefined subsets (i.e., innate and adaptive immunity activation, stress/inflammation) (Supplementary Table 1), none of these subsets were significantly associated with outcome at D90 (Figure 3B).

To explore immune restoration, the immune profiles at D7 were projected on the factorial map at D0 (Supplementary Figure 5), considering only individuals surviving at D7. A total of 39 individuals and 84 biomarkers were analyzed. Trajectories between D0 and D7 were clustered using k-means clustering. Results are reported in Figure 3C: each line represents the coordinate at D0 and at D7 for each individual. Their color depends on the cluster in which they are gathered.

The correlation circle for surviving patients at Day 7 as well as the variables with the strongest correlations (Supplementary Table 4) allows an interpretation of both clusters of trajectories. Horizontal lines in Figure 3C (red lines) and their average (in boldface) represent patients with stable immune profile trends between D0 and D7. These patients tended to have small values of biomarkers related to adaptive immune response and high values for biomarkers related to NK. On the other hand, a subgroup of patients (in black) showed decreasing trends between D0 and D7, hence their immune profile changed. These patients showed globally high values of biomarkers related to the adaptive immune response and low values for biomarkers related to NK at D0, while these values tended toward the average profile at D7 (in boldface).

These two groups of trajectories were not discriminative to predict survival at D90, suggesting that immune profile changes between D0 and D7 were not significantly associated with survival (Figure 3D).

### Immune Profile and Secondary Infections

Secondary infections were mostly bacterial infections. Patients who developed bacterial infections had lower CD8 lymphocytes (total, HLA-DR+/activated, effector and memory), B lymphocytes (transitional), γδ T lymphocytes and iNKT cells at admission compared to patients without bacterial infection (Supplementary Figure 6). The number of HLA-DR/monocytes was significantly lower by D7 but not at D0 in patients who developed bacterial infections. Other biomarkers were not statistically different between infected and non-infected patients.

**Figure 4:**
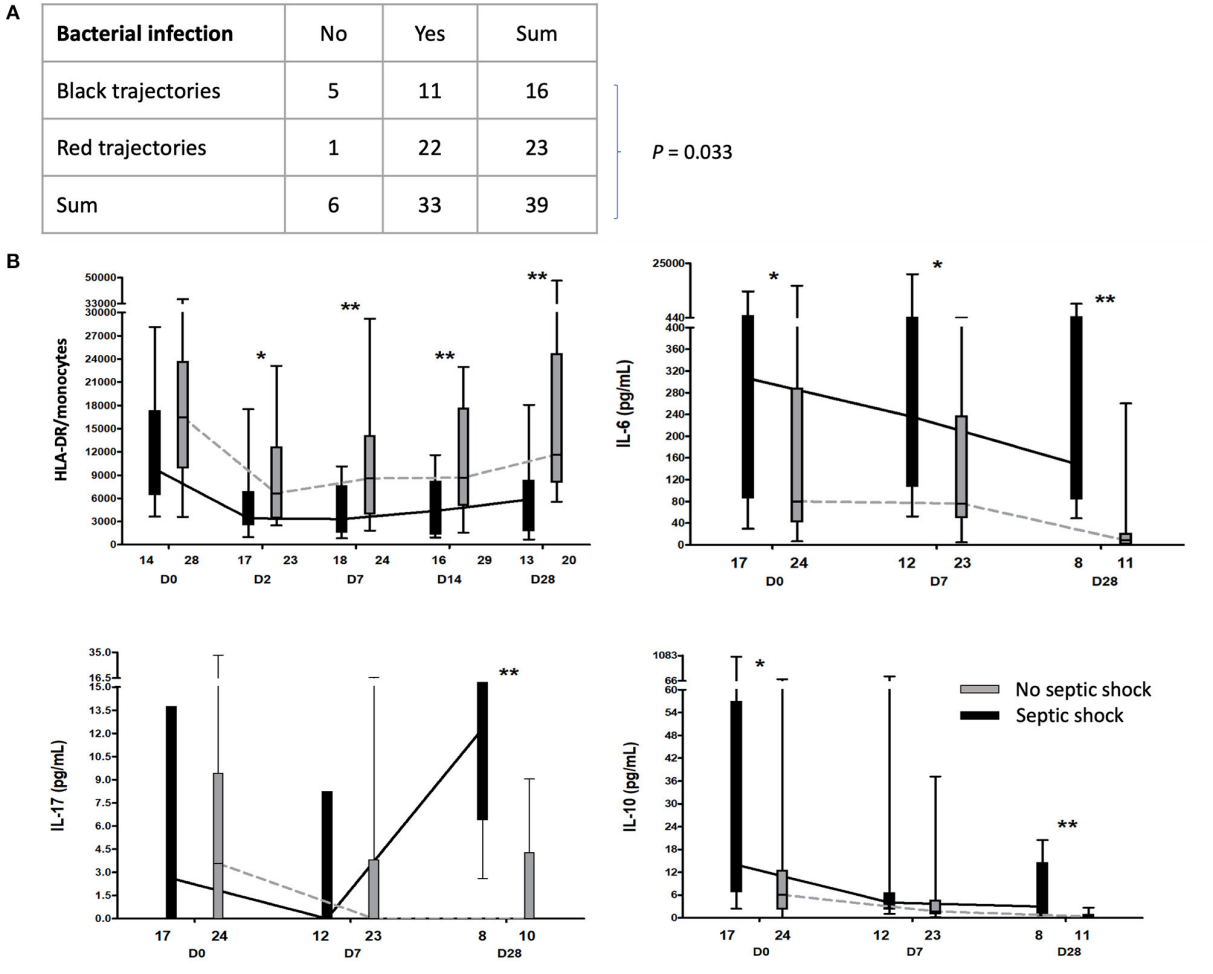
Association between immune profiles, bacterial infection and septic shock. **(A)** Contingency table displaying patients with red or black trajectory (refer to Figure 3C), depending on whether develop bacterial infection. **(B)** Kinetic analysis of the number of HLA-DR molecules per monocyte (HLA-DR/monocytes) and cytokines between patients with (in black) or without septic shock (in gray). *p < 0.05; **p < 0.001.

MFA did not show any clear cluster of immune responses during the first week associated with the risk of bacterial infection (Supplementary Figure 4B). However, immune profile trajectories between admission and D7 were associated with the risk of subsequent bacterial infections. Patients with positive projection on dimension 1 at admission and trending to zero at day 7 had lower incidence of bacterial infections (*p* = 0.033) (Figures 3C, 4A).

When focusing on patients with IFI, patients who later developed IFI showed lower HLA-DR/Monocytes and NK cell counts from D7 (Supplementary Figure 7). We did not find any biomarkers associated with the occurrence of viral reactivation.

Finally, patients who developed septic shock showed altered immune status compared to patients who did not develop septic shock, with sustained low HLA-DR/monocytes from D2 to D28 and high serum levels of IL-6, IL-10, and IL-17 at late time points (Figure 4B). Of note, MFA at D0 and D7 failed to show any differences between the two groups of patients (Supplementary Figure 4C).

## Discussion

In this study, we report the prospective and sequential immune profiles of 50 severely-ill burn patients, from the first hours after injury up to 28 days post-admission, analyzing how these associate with mortality at day 90.

Most, if not all, analyzed mediators of immunity pointed at overwhelming pro-inflammatory responses which may be related to the release of cell-derived damage associated molecular patterns (DAMPs) (18). These results suggest that patients at the time of admission already shared a common basal status combining monocyte dysfunction, T-cell activation, defects in innate immune responses which occurs within hours after the injury. However, no associations between immune profiles at D0 and outcomes, or between immune profile trajectories and outcome, have been highlighted.

We observed a significant decrease in iNKT, γδ-T and MAIT cell counts in blood during the initial response to burn injury. These cells, which are prompt responders to stress, self and foreign antigens, play a major role as a first line of defense and homeostasis in many tissues (19) and their disappearance from the blood may be related to re-localization within injured tissues. Looking into critical biomarkers of immune function and inflammation, some observations are worth mentioning. Patients displayed high absolute counts of circulating neutrophils and monocytes, which are critical cell subsets involved in acute inflammatory processes (20). Lower amounts of CD16 (FCgRIII receptor) were found at the cell surface of neutrophils. Receptors for IgG (FcgRs) are important triggers of numerous cellular effector functions and provide a link between the innate and adaptive immune responses. They allow phagocytosis, antibody-dependent cellular cytotoxicity (ADCC), and secretion of cytokines or other inflammatory mediators (21). Among them, CD16 (FcgRIIIB) and CD64 (FcgRI), were previously reported to be modulated during other inflammatory response conditions, e.g., sepsis (22). Cells in distress induce MICA on their cell surface and become NK-cell targets through the direct recognition by the activating NK receptor, NKG2D (23). Here, we found that NKG2D was significantly decreased on the cell surface of NK cells, potentially impairing their function. More surprisingly, an unexpected decrease of MICA expression on patients-derived neutrophils was also observed. Monocyte HLA-DR has been one of the most investigated biomarkers, showing in some cohorts that low expression identifies patients who are at higher risk of septic shock in intensive care units (9, 24, 25). We observed similar findings in our cohort. A down-regulation of HLA-DR expression was observed in our cohort, regardless of the severity of burn injury. HLA-DR is particularly sensitive to circulating levels of anti-inflammatory cytokines (1, 10), which were already dramatically up-regulated in the 50 patients at the early time-points from admission.

Our data showed that the pattern of the immune response at early time points (within the first week) was not significantly associated with mortality at day 90. Though, a delayed and sustained alteration of cytokine profiles and low HLA-DR/monocytes at late time points were more pronounced in non-survivors. Patients with the lowest counts of CD8 T-cells, γδ T-cells, B-cells and iNKT at the admission were more prone to bacterial infections. In addition, we found that inflammatory profiles between D0 and D7 were associated with risk of secondary bacterial infection. During time, we found that a delayed recovery of HLA-DR/monocytes and a late dysregulation of cytokine production were associated with the occurrence of septic shock. These data suggest that sustained alterations of immune and inflammatory response observed in the non-survivor group may be driven by the development of septic shock.

The overwhelming inflammatory processes have been previously proposed as a key contributors of organ injury and dysfunction in critically-ill patients, especially in sepsis (26). In addition, such immune profile may impair further orchestration of a proper immune response toward subsequent infections, with the occurrence of immune-paralysis (4). However, while the immune response was associated with risk of secondary infection, we did not observe a statistically significant association between immune response/restoration profiles and mortality at earliest time points. There are a series of factors that might explain our results. First, it could be argued that there is a lack of power in our cohort. While this is one of the largest exploratory cohorts of immune and inflammatory response after burn injury, the sample size remains low. Second, these results might challenge the view of a causal role of immune and inflammatory response in the death of critically-ill burn patients. The lack of statistical association also reflects a limited effect size of immune clusters and outcome in this cohort of very severely-ill patients. This illustrates that the outcome may be driven by factors and events way beyond the immunologic response to burn injury in this cohort. While the immunologic profile was associated with infections, the causative role of immune alteration and infection in the death may be questioned. Within our cohort, other factors including the severity of shock or acute respiratory distress syndrome, multiple organ failure with irreversible organ damage without recovery may have heavily contributed to death. The association between immune profile and outcome should next be explored in less severe patients. Interestingly, many trials failed to show an improvement of outcome after modulating the inflammatory response in sepsis or after cardio-pulmonary bypass. The association between inflammatory response, organ dysfunction and mortality has been heterogenous in burn injury patients. A systemic, intense and sustained inflammatory response was previously reported in pediatric patients that survive most often (27). Of note, our results revealed important differences in the immune and inflammatory responses across burn patients at admission. Despite lower total burn surface area, inflammatory responses are dampened in the elderly that have higher mortality than younger patients. Here, the median age was 49.2 years and only few elderly patients were included in our study. Burn patients showed higher cytokine levels than non-burn trauma patients, but no or poor association was found between multiple organ dysfunction or death and cytokine levels (28, 29). Several authors reported higher cytokine levels in non-survivors versus survivors (30–33). Stanojcic et al. reported a complex inflammatory and metabolic response to burn injury, associated with biomarkers of organ injury. Association with mortality was however not explored.

To conclude, in this cohort patients with the lowest counts of CD8 T-cells, γδ T-cells, B-cells and iNKT at the admission were more prone to bacterial infections. Furthermore, patients without bacterial infection displayed an early recovery pattern of both adaptive and innate immune responses. No cluster of immune/inflammatory biomarkers was associated with mortality, but later/delayed dysfunctional immune/inflammatory responses in non-survivors were compatible with the host response after development of septic shock (sustained alteration of cytokine profiles and low HLA-DR/monocytes at late time points). These results suggest that burn patients showed very early and profound immune and inflammatory alterations and that inability to restore their immune/inflammatory profile was associated with infections and poor outcomes.

## Data Availability Statement

The raw data supporting the conclusions of this article will be made available by the authors, without undue reservation.

## Ethics Statement

The studies involving human participants were reviewed and approved by ethical committee (IRB 00003835, protocol 2013/17NICB). The patients/participants provided their written informed consent to participate in this study.

## Author Contributions

HM-T and ML designed the study, collected the data, and drafted the manuscript. DC and QR collected the data, analyzed the data, and drafted the manuscript. VA conducted biostatistics analyses and drafted the manuscript. GM and LH conducted the assays and drafted the manuscript. MB, JL, and AT drafted the manuscript. All authors contributed to the article and approved the submitted version.

## Conflict of Interest

The authors declare that the research was conducted in the absence of any commercial or financial relationships that could be construed as a potential conflict of interest.

## Abbreviations

CM: central memory
EM: effector memory
EMRA: terminally differentiated memory
EORTC: European Organization for Research and Treatment of Cancer
HD: healthy donor
GLP: Good Laboratory Practice
HLA-DR/monocyte: number of HLA-DR molecules per monocyte
iNKT: invariant NKT
MAIT: Mucosal Associated Invariant T cell
MSG: Mycoses Study Group
PN: polynuclear cell
RTE: Recent Thymic Emigrant
Treg: regulatory T cell.
