# Supplementary material for: Severe Altered Immune Status After Burn Injury Is Associated With Bacterial Infection and Septic Shock
Source: Front Immunol. 2021 Mar 2;12:586195. doi: 10.3389/fimmu.2021.586195 (PMC7960913; doi:10.3389/fimmu.2021.586195)
Supplement: Supplementary file 1 [file Image_1.PDF]

Supplementary Figure 1: Flow Cytometry hierarchical gating strategy: Lymphocytes are identified using morphological (FSC/SSC) and CD45 staining strategies. Lymphocytes subsets are defined within parental or grand parental populations.
RTE: Recent Thymic Emigrant, CM: central memory, EM: effector memory, EMRA: terminally differentiated memory, Treg: regulatory T cells, MAIT: Mucosal-associated invariant T cells.

- All events
  - Polynuclear cells (PN: morphology and CD45+)
  - Monocytes (morphology and CD45+)
  - Lymphocytes (morphology and CD45+)
    - CD3+CD4+ (CD4+ T cells)
      - CD45RA+CCR7+ (Naive)
        - CD31+ (RTE)
      - CD45RA-CCR7+ (CM)
      - CD45RA-CCR7- (EM)
      - CD45RA+CCR7- (EMRA)
      - CD25hiCD127low (Treg)
      - CD25+
      - HLA-DR
      - CD279 (PD1)
    - CD3+CD8+ (CD84+ T cells)
      - CD45RA+CCR7+ (Naive)
      - CD45RA-CCR7+ (CM)
      - CD45RA-CCR7- (EM)
      - CD45RA+CCR7- (EMRA)
      - CD25+
      - HLA-DR
      - CD279 (PD1)
    - CD3+ (T cells)
      - NKG2D
    - CD3+ TCR $\gamma\delta$+
    - CD3+CD4-
      - CD161hiVα7.2+
    - CD3+iNKT+
    - CD3-(CD16+ and/or CD56+) (NK)
      - NKG2D
    - CD19+ (B cells)
      - CD27-IgD+
        - CD38+/-CD10- (Naive)
        - CD38hiCD10+ (Transitionnal)
      - CD27+IgD+ (Unswitched)
      - CD27-IgD-
        - CD38+/-CD10+/- (Memory)
        - CD38hi (Plasmocytes)
