# Supplementary material for: Severe Altered Immune Status After Burn Injury Is Associated With Bacterial Infection and Septic Shock
Source: Front Immunol. 2021 Mar 2;12:586195. doi: 10.3389/fimmu.2021.586195 (PMC7960913; doi:10.3389/fimmu.2021.586195)

Supplementary Figure 2: Flow cytometry representative examples in HD. A) Identification of PN, monocytes and Lymphocytes using both CD45 and morphological ciriteria**, B)** identification of Lymphocyte subsets: Naïve/memory subsets are defined within CD4+ and CD8+T cells as Naïve (CD45RA+CCR7+), CM (CD45RA-CCR7+), EM (CD45RA-CCR7-) and EMRA (CD45RA+CCR7-). Treg are defined as CD25hiCD127low within CD4+ T cells, RTE as CD31+ cells within the Naïve subset (CD45RA+CCR7+) of the grand parental CD3+CD4+ subset, MAIT as CD161hiVα7.2+ cells within the CD3+CD4- subset.

PN: Polynuclear cells, RTE: Recent Thymic Emigrant, CM: central memory, EM: effector memory, EMRA: terminally differentiated memory, Treg: regulatory T cells, MAIT: Mucosal-associated invariant T cells.

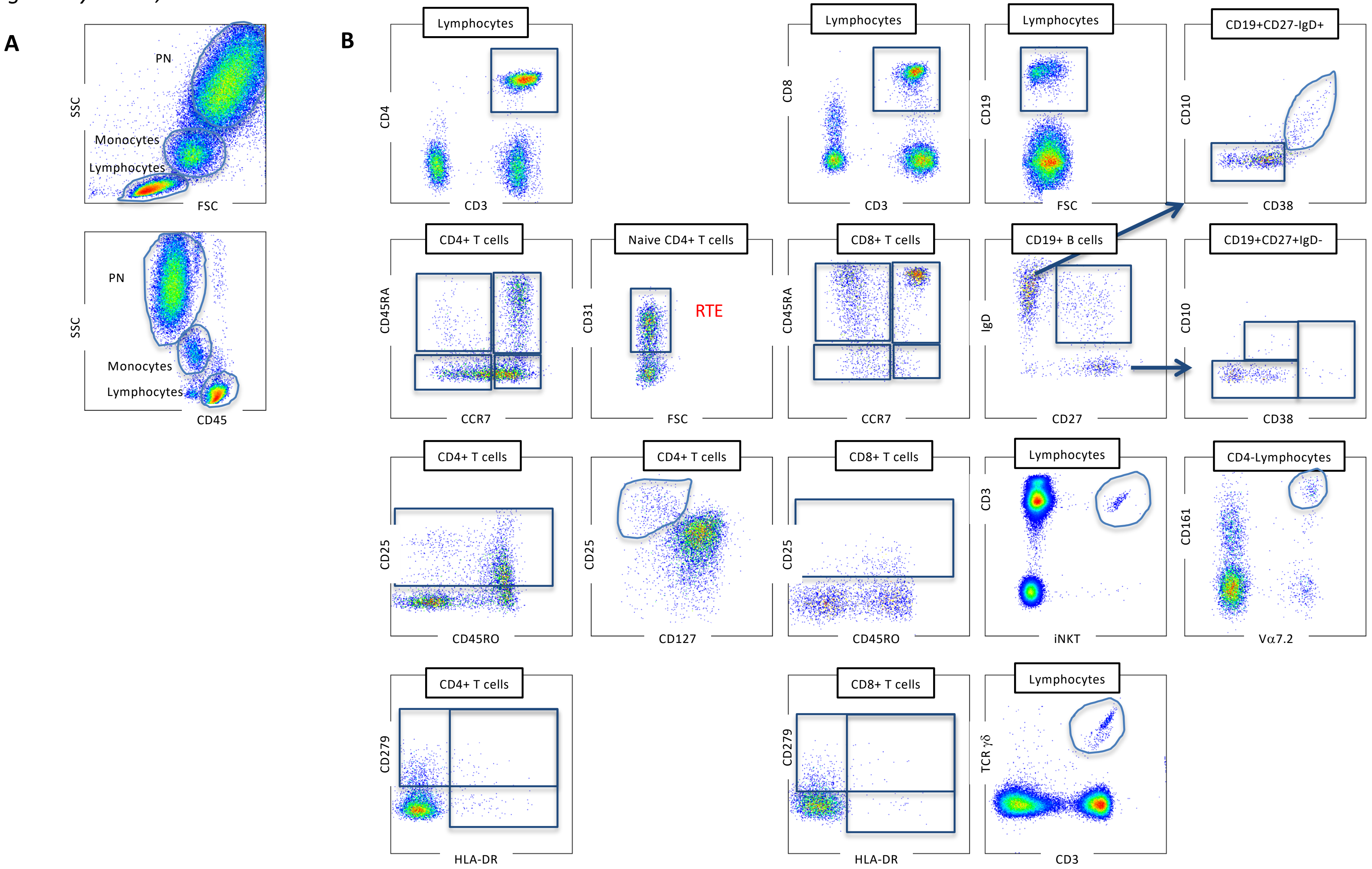

Supplement: Supplementary file 2 [file Image_2.PDF]
