# Supplementary material for: Severe Altered Immune Status After Burn Injury Is Associated With Bacterial Infection and Septic Shock
Source: Front Immunol. 2021 Mar 2;12:586195. doi: 10.3389/fimmu.2021.586195 (PMC7960913; doi:10.3389/fimmu.2021.586195)

Supplementary Figure 3: Flow cytometry representative examples in HD (red) and patients (blue). A/ HLA-DR cell-surface expression on monocytes, **B/**NKG2D cell-surface expression on NK cells, **C/** CD3 and iNKT percentages within the lymphocyte subset (left: HD, right: patient).

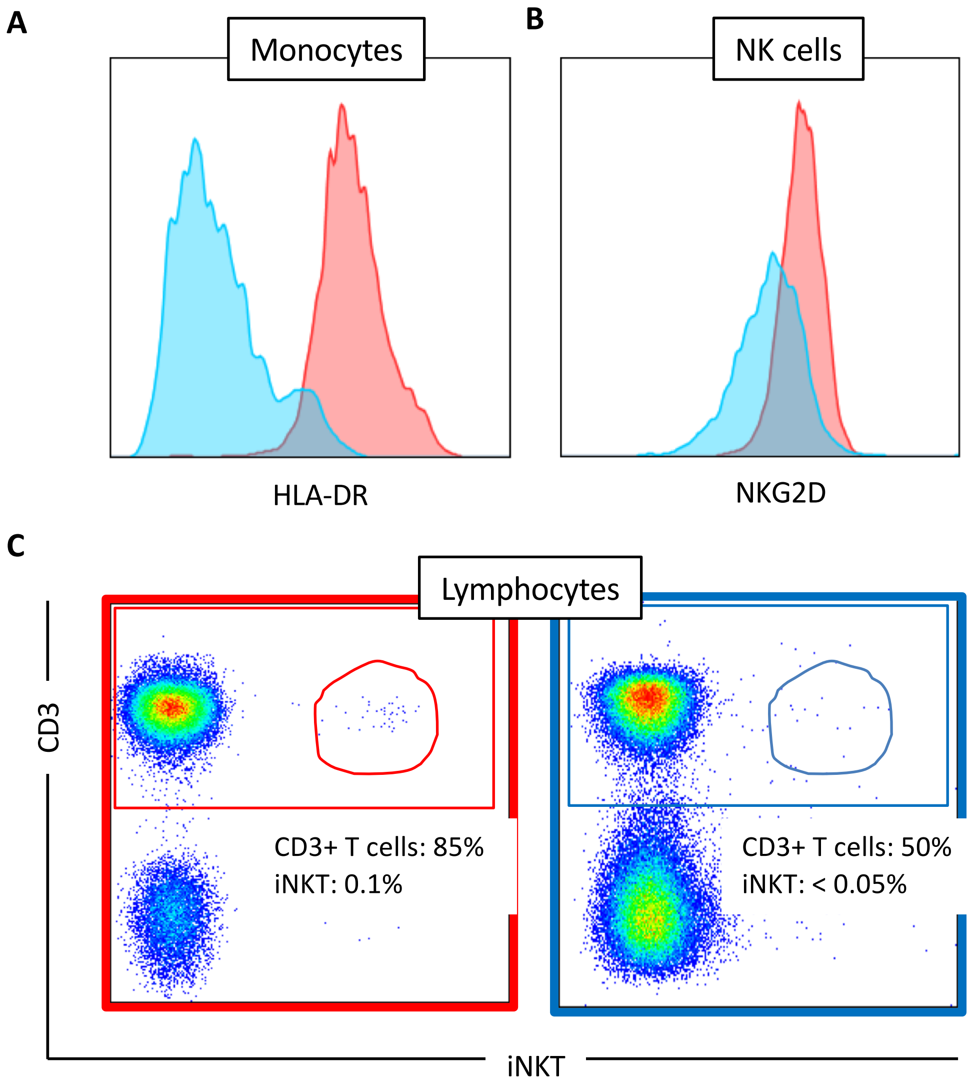

Supplement: Supplementary file 3 [file Image_3.PDF]
