# Supplementary material for: Severe Altered Immune Status After Burn Injury Is Associated With Bacterial Infection and Septic Shock
Source: Front Immunol. 2021 Mar 2;12:586195. doi: 10.3389/fimmu.2021.586195 (PMC7960913; doi:10.3389/fimmu.2021.586195)

Supplementary Figure 4: **Immune profiles and outcomes**: MFA is based on 84 biomarkers and 43 burn patients at day 7. The 4 groups of biomarkers at D7 are represented in the 2 main dimensions of the MFA. Prediction of survival at day 90 (A), infection (B) and septic shock occurrence (C). Black dots represent respectively alive patients at day 90 (A), non-bacterial infected patients (B) and no occurrence of septic shock (C) and red dots the opposite occurrence for each graph.

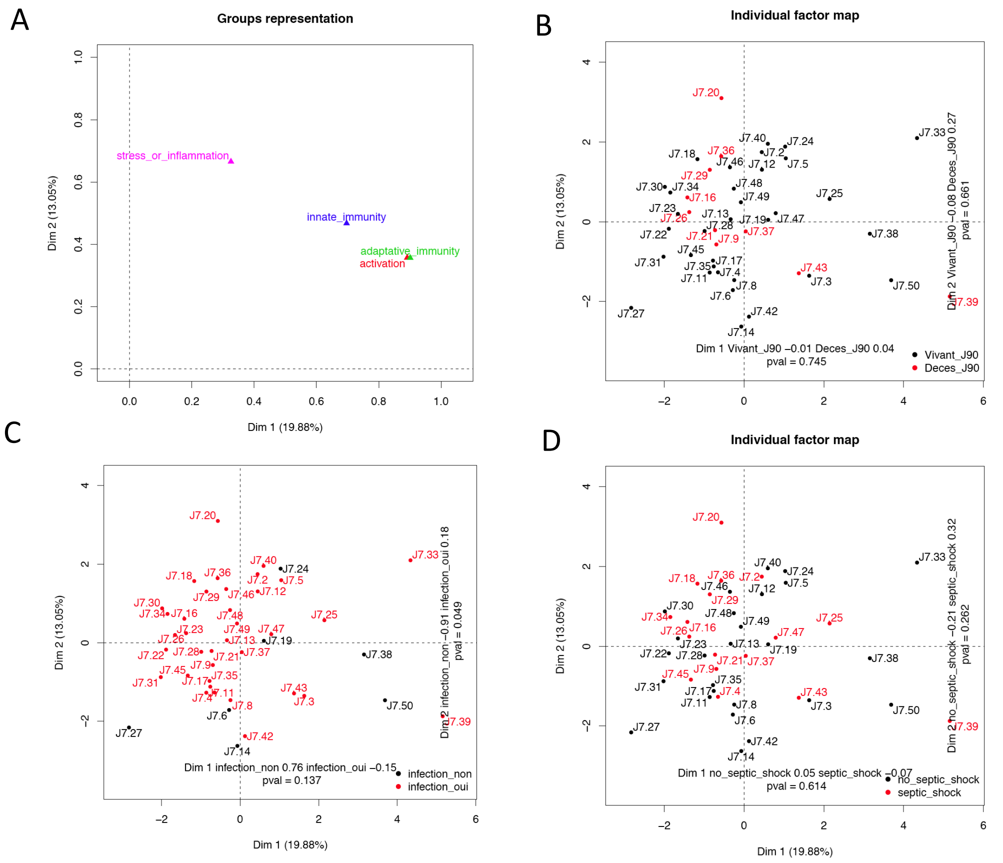

Supplement: Supplementary file 4 [file Image_4.PDF]
