# Supplementary figures and images for: Severe Altered Immune Status After Burn Injury Is Associated With Bacterial Infection and Septic Shock
Source: Front Immunol. 2021 Mar 2;12:586195. doi: 10.3389/fimmu.2021.586195 (PMC7960913; doi:10.3389/fimmu.2021.586195)

**Supplementary Figure 5: MFA on burn patients at D0: correlation circle**

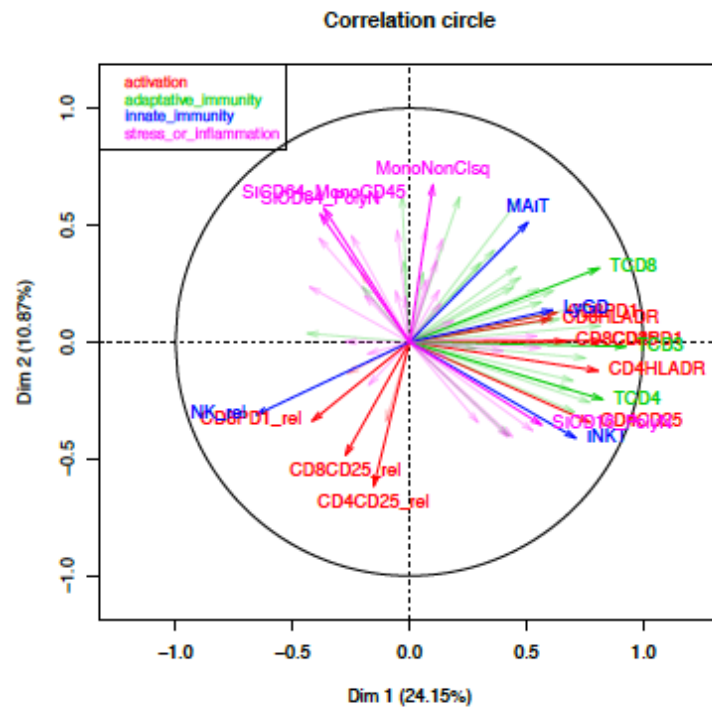

Supplement: Supplementary file 5 [file Image_5.PDF]

Supplementary Figure 6: Bacterial infection

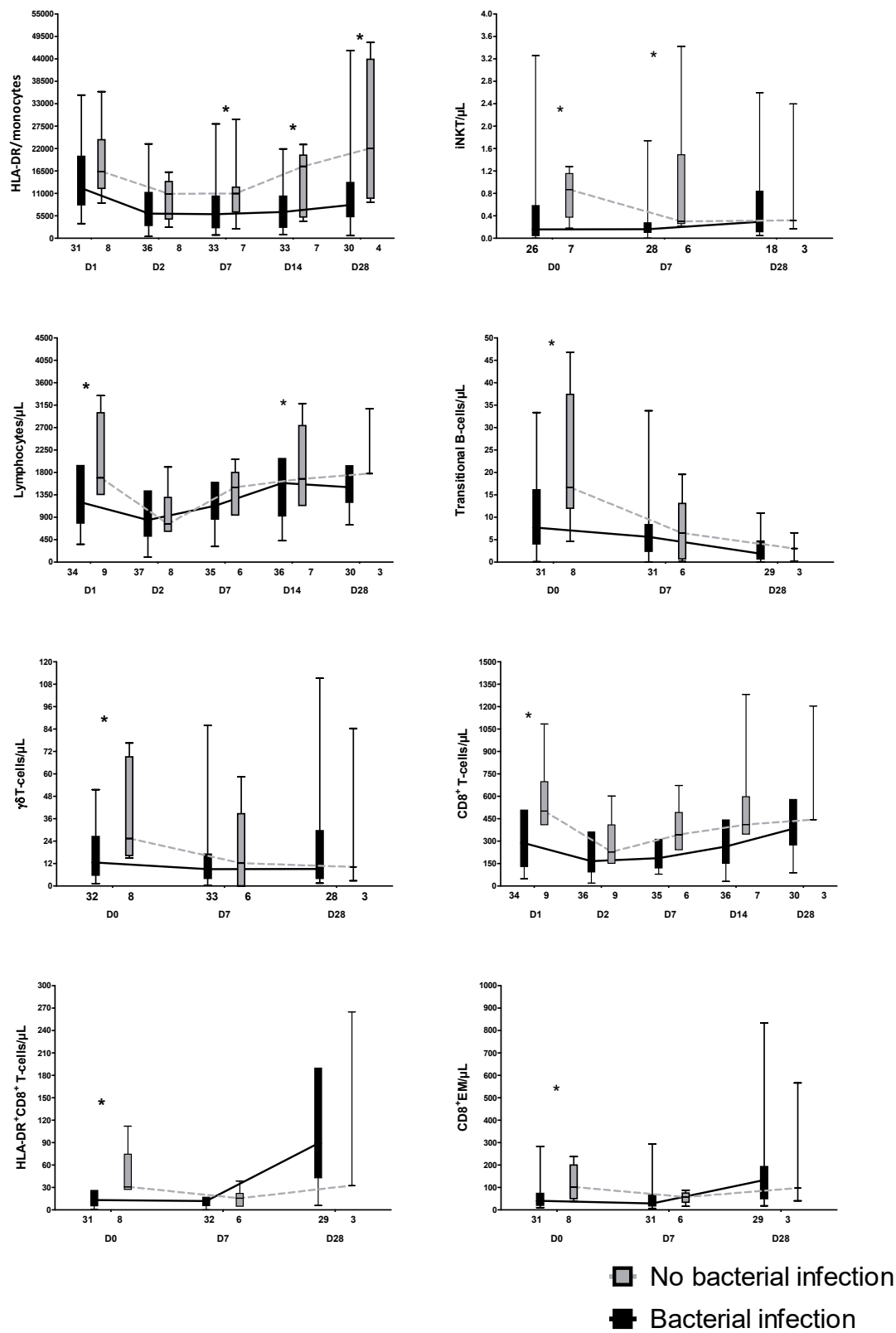

Supplement: Supplementary file 6 [file Image_6.PDF]

Supplementary Figure 7: Fungal infections

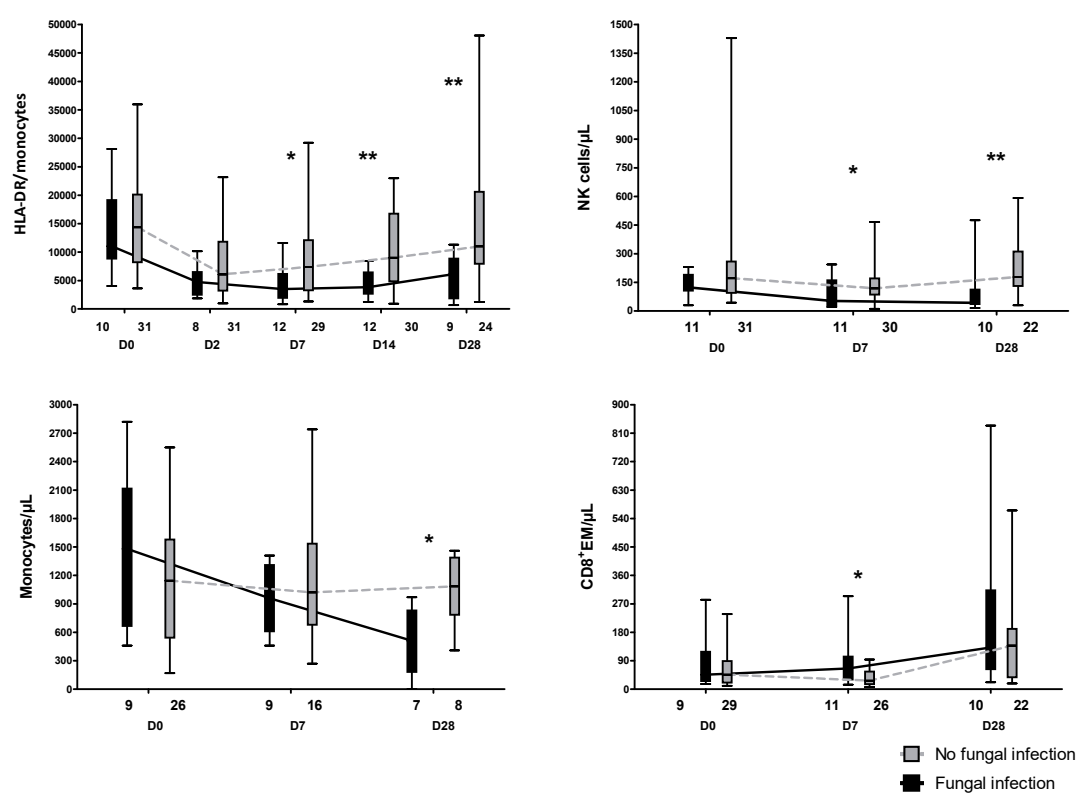

Supplement: Supplementary file 7 [file Image_7.PDF]

Supplementary Figure 8: Viral infections

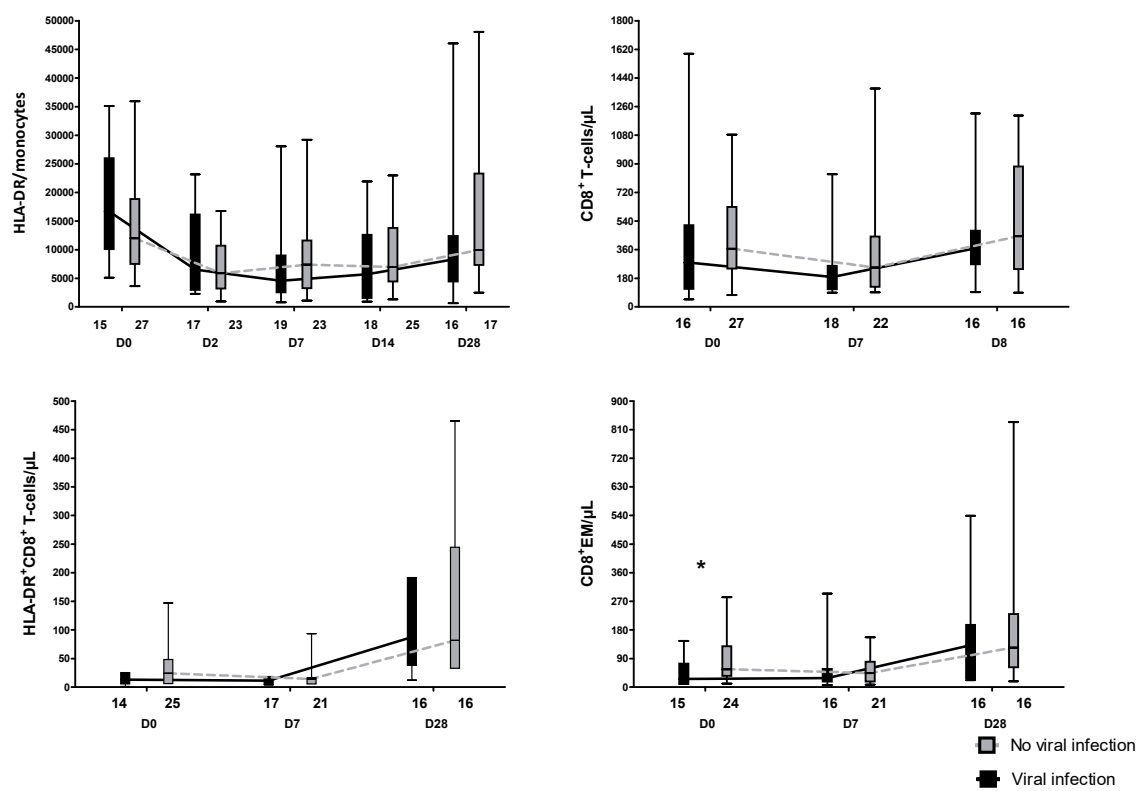

Supplement: Supplementary file 8 [file Image_8.PDF]
