# Supplementary material for: Severe Altered Immune Status After Burn Injury Is Associated With Bacterial Infection and Septic Shock
Source: Front Immunol. 2021 Mar 2;12:586195. doi: 10.3389/fimmu.2021.586195 (PMC7960913; doi:10.3389/fimmu.2021.586195)
Supplement: Supplementary file 9 [file Table_1.DOCX]

**Supplementary Table 1**: *Abs: absolute, CM: Central Memory, EM: Effector Memory, EMRA: terminally differentiated, Treg: regulatory T cell, RTE: recent thymic emigrants*
