# Supplementary material for: Severe Altered Immune Status After Burn Injury Is Associated With Bacterial Infection and Septic Shock
Source: Front Immunol. 2021 Mar 2;12:586195. doi: 10.3389/fimmu.2021.586195 (PMC7960913; doi:10.3389/fimmu.2021.586195)
Supplement: Supplementary file 10 [file Table_2.DOCX]

**Supplementary Table 2: MFA on burn patients and healthy donors:** list of biomarkers with the highest coordinates on dimensions 1 and 2
