# Supplementary material for: Severe Altered Immune Status After Burn Injury Is Associated With Bacterial Infection and Septic Shock
Source: Front Immunol. 2021 Mar 2;12:586195. doi: 10.3389/fimmu.2021.586195 (PMC7960913; doi:10.3389/fimmu.2021.586195)
Supplement: Supplementary file 11 [file Table_3.DOCX]

**Supplementary Table 3: MFA on burn patients at D0:** list of biomarkers with the highest coordinates on dimension 1

| **Dimension 1** | |
| --- | --- |
| **Biomarker** | **correlation** |
| CD3+ T-cells/uL | 0.92 |
| CD4+T-cells/uL | 0.83 |
| CD8+T-cells/uL | 0.81 |
| CD8+Naive T-cells/uL | 0.76 |
| PD1+CD4+T-cells/uL | 0.73 |
| HLA-DR+CD8+T-cells/uL | 0.72 |
| CD4+CM T-cells/uL | 0.71 |
| CD25+CD4+T-cells/uL | 0.69 |
| RTE/uL | 0.69 |
| CD4+EM T-cells/uL | 0.69 |
| Treg/uL | 0.69 |
| Naive B-cells /uL | 0.69 |
| NK% /Lymphocytes | -0.65 |
